# The role of dietary sodium intake on the modulation of T helper 17 cells and regulatory T cells in patients with rheumatoid arthritis and systemic lupus erythematosus

**DOI:** 10.1371/journal.pone.0184449

**Published:** 2017-09-06

**Authors:** Rossana Scrivo, Laura Massaro, Cristiana Barbati, Marta Vomero, Fulvia Ceccarelli, Francesca Romana Spinelli, Valeria Riccieri, Alessandra Spagnoli, Cristiano Alessandri, Giovambattista Desideri, Fabrizio Conti, Guido Valesini

**Affiliations:** 1 Department of Internal Medicine and Medical Specialties, Rheumatology; Sapienza University of Rome, Rome, Italy; 2 Department of Public Health and Infectious Diseases, Sapienza University of Rome, Rome, Italy; 3 Geriatric Unit, Department of Life, Health and Environmental Sciences, University of L'Aquila, L'Aquila, Italy; Keio University, JAPAN

## Abstract

We aimed at investigating whether the frequency and function of T helper 17 (Th17) and regulatory T cells (Treg) are affected by a restriction of dietary sodium intake in patients with rheumatoid arthritis (RA) and systemic lupus erythematosus (SLE). We enrolled RA and SLE patients not receiving drugs known to increase urinary sodium excretion. Patients underwent a dietary regimen starting with a restricted daily sodium intake followed by a normal-sodium daily intake. The timepoints were identified at baseline (T0), after 3 weeks of low-sodium dietary regimen (T3), after 2 weeks of normal-sodium dietary regimen (T5). On these visits, we measured the 24-hour urinary sodium excretion, the frequency and function of Th17 and Treg cells in the peripheral blood, the serum levels of cytokines. Analysis of urinary sodium excretion confirmed adherence to the dietary regimen. In RA patients, a trend toward a reduction in the frequencies of Th17 cells over the low-sodium dietary regimen followed by an increase at T5 was observed, while Treg cells exhibited the opposite trend. SLE patients showed a progressive reduction in the percentage of Th17 cells that reached a significance at T5 compared to T0 (p = 0.01) and an increase in the percentage of Treg cells following the low-sodium dietary regimen at both T1 and T3 compared to T0 (p = 0.04 and p = 0.02, respectively). No significant apoptosis or proliferation modulation was found. In RA patients, we found a reduction at T5 compared to T0 in serum levels of both TGFβ (p = 0.0016) and IL-9 (p = 0.0007); serum IL-9 levels were also reduced in SLE patients at T5 with respect to T0 (p = 0.03). This is the first study investigating the effects of dietary sodium intake on adaptive immunity. Based on the results, we hypothesize that a restricted sodium dietary intake may dampen the inflammatory response in RA and SLE patients.

## Introduction

Rheumatoid arthritis (RA) and systemic lupus erythematosus (SLE) are chronic autoimmune diseases sustained by impaired immunoregulatory processes alongside environmental factors in genetically susceptible individuals [1]. Among the environmental factors, hormones, cigarette smoking, and microbial agents have been implicated as favouring incident cases of both diseases; in addition, silica exposure and excess body mass have been considered for RA and exposure to ultraviolet light for SLE [2].

More recently, the role of dietary factors has gained interest due to the results of 2 independent studies, in which a modulation in T cells immune response was observed after a few weeks in murine models of autoimmune diseases following an excess salt intake [3,4]. Both studies demonstrated that high concentrations of sodium chloride promote the differentiation of T helper (Th) lymphocytes toward the Th17 phenotype, known to be highly pro-inflammatory [5], and the Th17-modulating effects of sodium chloride were found to be critical for the development of experimental autoimmune encephalomyelitis, an animal model for multiple sclerosis (MS) [3].

Up to now, only 2 studies in humans aimed at ascertain whether a surplus in sodium dietary intake may affect the emergence of autoimmune diseases [6,7]. In the former, a nested case-control study was set up in patients with RA to take advantage of the data obtained since 1991 among the citizens of a county in northern Sweden, who had been invited to participate in a screening and intervention programme for risk factors for cardiovascular diseases. Although no significant association was found between sodium intake and the development of RA, in further analyses adjusting for defined risk factors for RA significant associations were observed among smokers, in whom sodium intake more than doubled the risk of developing RA. Additive interaction analyses suggested that approximately half of the amount of risk from smoking in the development of RA was due to interaction with sodium intake [6]. In the second study, sodium intake was estimated from urinary sodium excretion in 70 patients with relapsing-remitting MS observed for 2 years. Not only was a positive correlation found between exacerbation rate and sodium intake in a multivariate model but, interestingly, individuals with high-sodium intake had a greater chance of developing a new lesion on the brain and spinal cord magnetic resonance imaging. However, a clear association between dietary sodium intake and disease activity could not be claimed because the cohort size was relatively small, the serum sodium levels remained rather constant under different dietary conditions, and the exclusion of confounders was not possible [7].

Overall, both experimental and clinical studies support the possibility that a high dietary sodium intake may promote the pro-inflammatory response in autoimmune diseases, possibly via the activation of Th17 lymphocytes. It was believed that Th17 cells were functionally antagonists to regulatory T cells (Treg), which are pivotal for controlling autoimmunity, and the dichotomy was also extended to their generation [8]. This view has subsequently changed, when studies revealed that Th17 and Treg cells may develop from the same precursors under distinct cytokine conditions [9] and a subset of IL-17-producing Treg cells can be generated upon polarization by cytokines [10].

Since no data are available on the biological effects of excess sodium in patients with autoimmune diseases, the aim of the present study was to investigate whether the frequency and function of Th17 and Treg cells are affected by a restriction of dietary sodium intake in RA and SLE patients observed over a five-week period.

## Patients and methods

### Patient population

The study received Policlinico Umberto I Ethics Committee approval in accordance with local requirements (prot. n. 1256/14) and written informed consent was obtained from each participant. Between October 2014 and March 2016, we enrolled consecutive Caucasian RA and SLE patients fulfilling the ACR/EULAR 2012 [11] and ACR 1997 [12] classification criteria, respectively. A prednisone dose of >5 mg/day or equivalent was considered as an exclusion criterion based on the findings that glucocorticoids increase urine volume, promote urinary sodium and potassium excretion, and affect the capability of mineralcorticoids to retain sodium [13]. Likewise, none of the patients were taking other drugs known to increase urinary sodium excretion including thiazide diuretics, potassium-sparing diuretics, loop diuretics, angiotensin converting enzyme-inhibitors, and angiotensin II AT1 receptor antagonists [14–16]. Other treatments, including immunosuppressants, were allowed provided that were taken at a stable dose for at least 4 weeks prior to the baseline visit and patients were asked to remain on a stable dose throughout the study period. None of them were on a weight-loss diet and/or used dietary supplements.

### Study design

The study was planned to cover a five-week period and has a within-subjects design in which all of the participants served as their own control. Given the harmful effects of excess sodium consumption [17], patients underwent a dietary regimen starting with a restricted daily sodium intake followed by a normal-sodium daily intake, thus avoiding the exposure to a surplus in sodium dietary intake. The first week served as the “run-in” period, allowing patients to get used to the low-sodium diet following the indications in the leaflet given to each of them (Supplementary Material). The intent was to measure the adherence to the diet and the countercheck was provided by testing the 24-hour urine excretion of sodium at the end of the week. This was supposed to be <85 mEq/die, based on the assumption that a low-sodium dietary regimen is defined by <5 grams of sodium chloride a day [18] and that 1 gram of sodium chloride contains 17 mEq of sodium [19]. If the 24-hour urinary sodium excretion target was met, patients were asked to continue the low-sodium diet regimen for other 2 weeks and, again, another control of urinary sodium excretion was performed to check for the adherence to diet. Finally, patients entered the last 2 weeks of the study, which were devoted to a normal-sodium dietary regimen. To achieve this goal, they were given a total of 70 bags of cooking sodium (each containing 1 gram of sodium) to enrich their low-sodium intake diet (5 grams a day). At the end of the study, the 24-hour urinary sodium excretion was determined.

The main timepoints of the study were identified at baseline, before starting the low-sodium dietary regimen (T0); after 3 weeks, at the end of low-sodium dietary regimen (T3); at the end of the study, after 2 weeks of normal-sodium dietary regimen (T5). As mentioned, on these visits we measured the 24-hour sodium excretion in urine, which is considered the most reliable method for measuring urinary sodium excretion and an accepted estimate of sodium intake [20,21]. Furthermore, patients underwent venous blood sampling to analyze the frequency of Th17 and Treg cells as well as their function through analysis of apoptosis and proliferation; serum was also obtained for cytokine measurements.

### Preparation of PBMCs and flow cytometry analysis to identify Th17 and Treg cells

PBMCs were isolated by Ficoll-Hypaque density-gradient centrifugation [22] to identify Th17 cells, defined as IL-17 expressing CD4+ T cells [23], and Treg cells, defined as CD4+FoxP3+ T cells [24]. Surface and intracellular phenotyping of PBMCs was performed with combinations of fluorescein isothiocyanate (FITC), phycoerythrin (PE), peridinin chlorophyll protein (PerCP) or allophycocyanin (APC)-labelled monoclonal antibodies (mAbs) as described before [25]. For surface staining, conjugated mAbs against human CD4, CD45RA and CD25 (all from BD Biosciences, San Jose, CA, USA) were used. The intracellular detection of IL-17, FoxP3 and Ki-67 with PerCP-labelled anti-IL-17 (clone eBio64DEC17; eBioscience), PE-labelled anti-FoxP3 (clone 236A/E7; eBioscience), and PerCP-labelled anti-Ki-67 (BD Biosciences) was obtained on cells fixed and permeabilized using Fix/Perm solution (eBioscience). For the detection of intracellular IL-17 production, PBMCs were stimulated for 4 hours with 50 ng/ml phorbol 12-myristate 13-acetate (PMA) and 1 μg/ml ionomycin in the presence of 10 μg/ml Brefeldin A (all from Sigma-Aldrich, St. Louis, MO, USA), and then stained with PerCP-labelled anti-IL-17 mAb after fixation and permeabilization. Among Treg cells, we measured the 3 functionally distinct subsets that were recently recognized [24]: the naïve or resting Treg cells (nTreg), defined by the CD4+CD45RA+FoxP3^**low**^ phenotype; the effector or activated Treg cells (eTreg), defined by the CD4+CD45RA-FoxP3^**high**^ phenotype; the FoxP3 expressing non-regulatory Treg cells (non-Treg), defined by the CD4+CD45RA-FoxP3^**low**^ phenotype, which exhibit the capability to trans-differentiate into Th17 cells in the presence of a favourable local cytokine milieu.

### Preparation of PBMCs to detect apoptosis and lymphocytes proliferation

**Fig 1:**
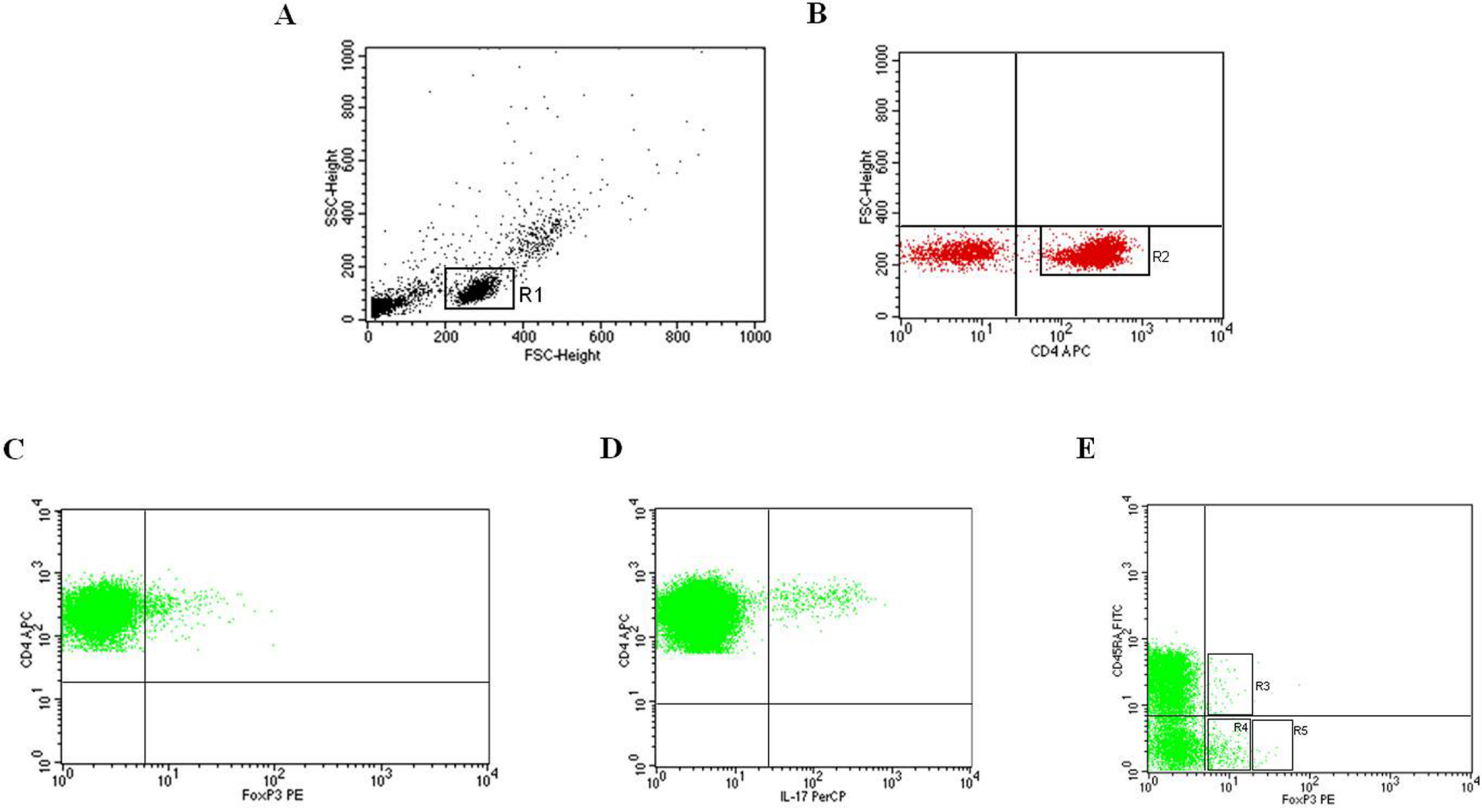
Representative dot plots of peripheral blood lymphocytes from one patient showing the gating strategy to identify Th17 and Treg cells. (A) Live cells, gated for cell death and debris exclusion, were then gated as lymphocytes (R1) based on morphological parameters (SSC, side scatter; FSC, forward scatter). (B) Identification of CD4+ lymphocytes (R2) within the R1 population. (C) Identification of Treg cells and (D) Th17 cells within the R2 population. (E) Identification of the I, II and III fractions of Treg cells within the R2 population. The values were expressed as percentage of CD4+ T cells.

Spontaneous apoptosis of PBMCs was measured immediately following lymphocyte separation (ex vivo apoptosis) using FITC-conjugated annexin V (AV) and propidium iodide (PI) apoptosis detection kit (Marine Biological Laboratory, Woods Hole, MA) according to the manufacturer's protocol. The combination of FITC-conjugated AV and PI allows the differentiation among early apoptotic cells (AV+PI-), late apoptotic cells (AV+PI+), dead cells (AV-PI+), and total apoptosis (AV++, including early and late apoptotic cells). Finally, the proliferation of CD4+ T lymphocytes was evaluated by measuring the expression of the human Ki-67 protein, which is considered an excellent marker for determining the growth fraction of a cell population because it is present during all active phases of the cell cycle and mitosis but it is absent from resting cells [26]. Cells were detected by sequential gating for CD4+, CD45RA+, and FoxP3^low/high^. For gating strategy of the analysis of Th17 and Treg cells, see Fig 1. Acquisition was performed on a FACSCalibur cytometer (BD Immunocytometry Systems), and the data were analyzed using CellQuest Pro software (BD Immunocytometry Systems, San Jose, CA).

### Enzyme-linked immunosorbent assay (ELISA) to detect serum cytokines

Sera were obtained at T0, T3 and T5 by standard methods and stored at **-**80°C until used. We decided to measure the levels of IFNα, TGFβ1, IL-23, IL-1β, IL-6, IL-9, IL-12, IL-17, and TNF, due to their known pro-inflammatory/immunoregulatory properties. Serum IFNα concentrations were determined using a commercially available ELISA kit (R&D Systems, Minneapolis, MN) following the manufacturer’s instructions. The other cytokines were assessed using a cytokine panel by Bio-Plex assays (Bio-Rad Laboratories, Richmond, CA, USA) following the manufacturer's instructions. Data were analyzed using the Bio-Plex Manager software, version 4.1.1 (Bio-Rad Laboratories), reported as fluorescence intensity (FI) and subsequently converted into concentration (pg/ml).

### Statistical analysis

Data are expressed as median/25^th^-75^th^ percentile or percentages when appropriate. The comparison of percentages was performed using the χ^2^ test or Fisher's exact test when appropriate. The comparisons for continuous variables between patients with RA and SLE was made with the Mann-Whitney test, while multiple comparisons in patients at different times was made with the Friedman test with Dunn correction for dependent samples. The significance of any correlation was determined by Spearman’s rank correlation coefficient. P values <0.05 indicated statistical significance.

Statistical analysis was performed using GraphPad Prism 6 software (GraphPad Software, San Diego, CA, USA).

## Results

### Clinical and demographic characteristics of patient population

The clinical and demographic characteristics of patients with RA and SLE are presented in Tables 1 and 2, respectively. Overall, 60 patients were approached, but only 30 (15 with RA and 15 with SLE) were willing to participate and signed the informed consent; among these, one patient with RA failed to undergo the low sodium dietary regimen and withdrew after 3 weeks. Demographic parameters were balanced between the 2 groups with the exception of age, which was significantly higher in patients with RA with respect to those with SLE (p = 0.0008). Disease activity was evaluated at baseline in patients with RA by DAS28 [27], and in patients with SLE by SLEDAI-2K [28]. The disease activity was low in accordance with the need to take a low dose of glucocorticoids for patients to be enrolled and, based on the short duration of the study and on stable treatment during follow-up, we decided not to reevaluate disease activity at the end of the study.

**Table 1:** Demographic, clinical, serological and therapeutic features of patients with rheumatoid arthritis (n = 14).

Females, no.	13
Age, median/25°-75° percentile years	58.5 (50–67)
Disease duration, median/25°-75° percentile months	72 (33–225)
DAS28 by ESR, median/25°-75° percentile	3.2 (2.4–3.8)
**SEROLOGICAL FEATURES, no. (%)**	
RF+	10 (71.4)
ACPA+	6 (42.8)
ACPA+/RF+	6 (42.8)
ACPA-/FR-	4 (28.6)
ESR, median/25°-75° percentile mm/h	13 (6.5–19.2)
CRP, median/25°-75° percentile mg/dl	0.05 (0–0.3)
**CIGARETTE SMOKING, no. (%)**	
Smokers	2 (14.3)
Non-smokers	7 (50)
Ex-smokers	5 (35.7)
**CONCOMITANT TREATMENT**	** **
Glucocorticoids, no. (%)	6 (42.8)
Weekly dose of PDN, median/25°-75° percentile mg	0/0-35
DMARDs, no. (%)	7 (50)
Biologic DMARDs, no. (%)	7 (50)

DAS28 = disease activity score; ESR = erythrocyte sedimentation rate; RF = rheumatoid factor; ACPA = anticitrullinated protein antibody; CRP = C-reactive protein; PDN = prednisone; DMARDs = disease-modifying antirheumatic drugs.

**Table 2:** Demographic, clinical, serological and therapeutic features of patients with systemic lupus erythematosus (n = 15).

Female, no.	15
Age, median/25°-75° percentile years	44 (30–50)
Disease duration, median/25°-75° percentile months	108 (48–264)
**CLINICAL FEATURES, no. (%)**	
Skin	6 (40)
Joint	14 (93.3)
Serositis	1 (6.7)
Hematological	8 (53.3)
Renal	3 (20)
NPSLE	0
**SEROLOGICAL FEATURES, no. (%)**	
ANA	15 (100)
Anti-dsDNA	9 (60)
Anti-ENA	7 (46.7)
Anti-SSA	6 (40)
Anti-SSB	1 (6.7)
Anti-Sm	1 (6.7)
Anti-RNP	1 (6.7)
anti-CL IgG/IgM	7 (46.7)
anti-β2GPI IgG/IgM	5 (33.3)
LAC	3 (20)
Low C3 and C4 level	9 (60)
**CLINIMETRICS**	
SLEDAI-2K, median/25°-75° percentile	2 (0–4)
**CIGARETTE SMOKING, no. (%)**	
Smokers	3 (20)
Non-smokers	8 (53.3)
Ex-smokers	4 (26.7)
**CONCOMITANT TREATMENT**	** **
Glucocorticoids, no. (%) Weekly dose of PDN, median/25°-75° percentile mg	6 (40) 0/0-10
Hydroxychloroquine, no. (%)	12 (80)
Immunosuppressive drugs, no. (%)	3 (20)
Anti-BLyS, no. (%)	2 (13.3)

NPSLE: neuropsychiatric systemic lupus erythematosus; ANA = antinuclear antibodies; Anti-dsDNA = anti-double-stranded DNA; LAC = lupus anticoagulant; C3 = complement fraction 3; C4 = complement fraction 4; SLEDAI-2K = Systemic Lupus Erythematosus Disease Activity Index 2000; PDN = prednisone; Anti-BLyS = anti-B-lymphocyte stimulator.

### Adherence to the dietary regimen according to the study design

**Fig 2:**
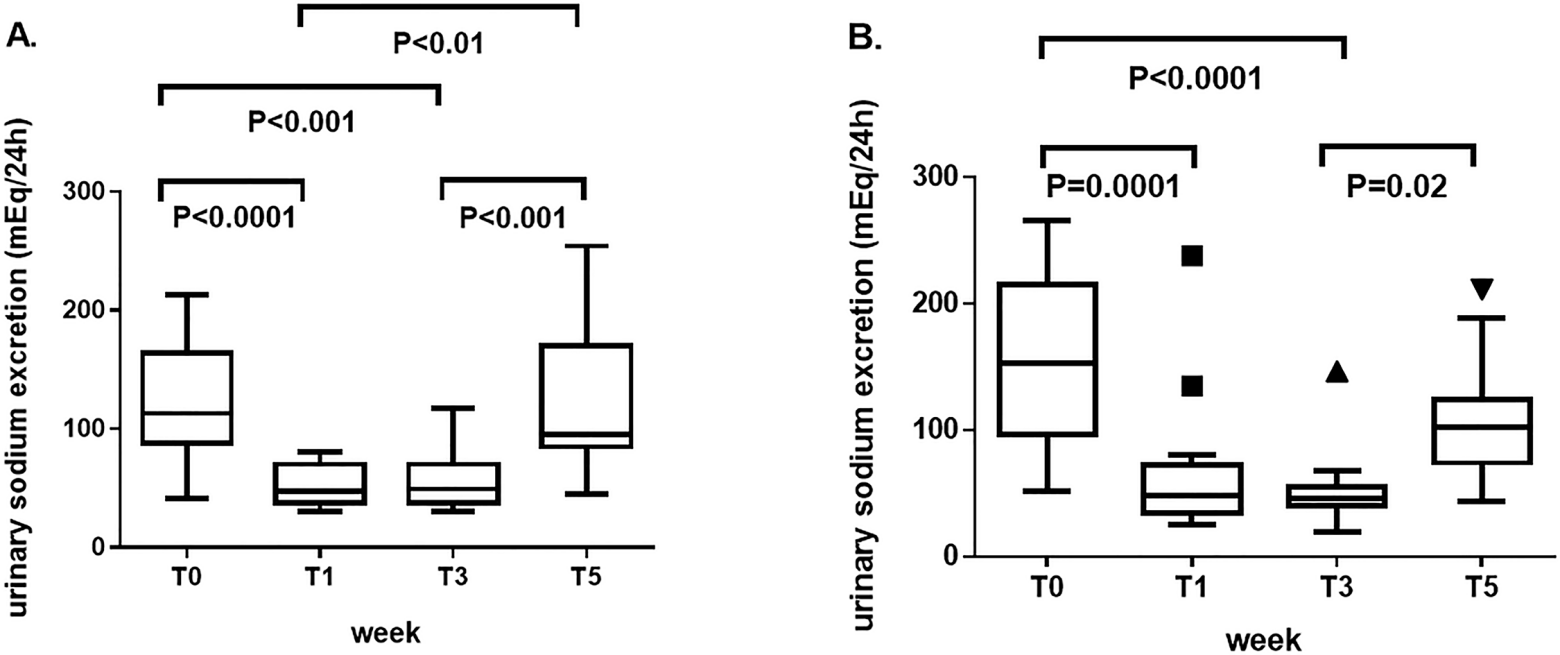
Comparison of urinary sodium excretion (mEq/24h) between the main timepoints of the study set at T0 (baseline, before starting the low-sodium dietary regimen), T1 (after 1 week, at the end of the “run-in” period of low-sodium dietary regimen), T3 (after 3 weeks, at the end of low-sodium dietary regimen), and T5 (at the end of the study, after 2 weeks of normal sodium dietary regimen). (A) In patients with rheumatoid arthritis, levels of 24-hour urinary sodium excretion significantly decreased from T0 to T1 and from T0 to T3 (after 3 weeks, at the end of low-sodium dietary regimen), and increased from T1 to T5 (at the end of the study, after 2 weeks of normal sodium dietary regimen) and from T3 to T5. (B) In patients with systemic lupus erythematosus, levels of 24-hour urinary sodium excretion significantly decreased from T0 to T1 and from T0 to T3, and increased from T3 to T5. Data are shown as Tukey boxplots; lines represent the median level with 25^th^-75^th^ percentile; data not included between the whiskers are plotted as outliers with dots.

To prove the adherence to the diet, 24-hour urinary sodium excretion was measured at T0, T1, T3, and T5. The results confirmed the correct attitude of the patients to the new dietary regimen throughout the study. In patients with RA, levels of urinary sodium excretion (median/25^th^-75^th^ percentile) significantly decreased from T0 (113.5/84.9–165.5) to T1 (47.5/36.9–71.3; p<0.0001) and from T0 to T3 (49.6/36.6–72; p<0.001), and increased from T1 to T5 (95.5/82.1–170.7; p<0.01) and from T3 to T5 (p<0.001). In patients with SLE, levels of urinary sodium excretion significantly decreased from T0 (153/96.3–215) to T1 (48/35-72.7; p = 0.0001) and from T0 to T3 (46/40.3–55; p<0.0001), and increased from T3 to T5 (102/74.4–124.3; p = 0.02) (Fig 2A and 2B).

### Change of Th17 and Treg cell frequencies alongside dietary regimen

**Fig 3:**
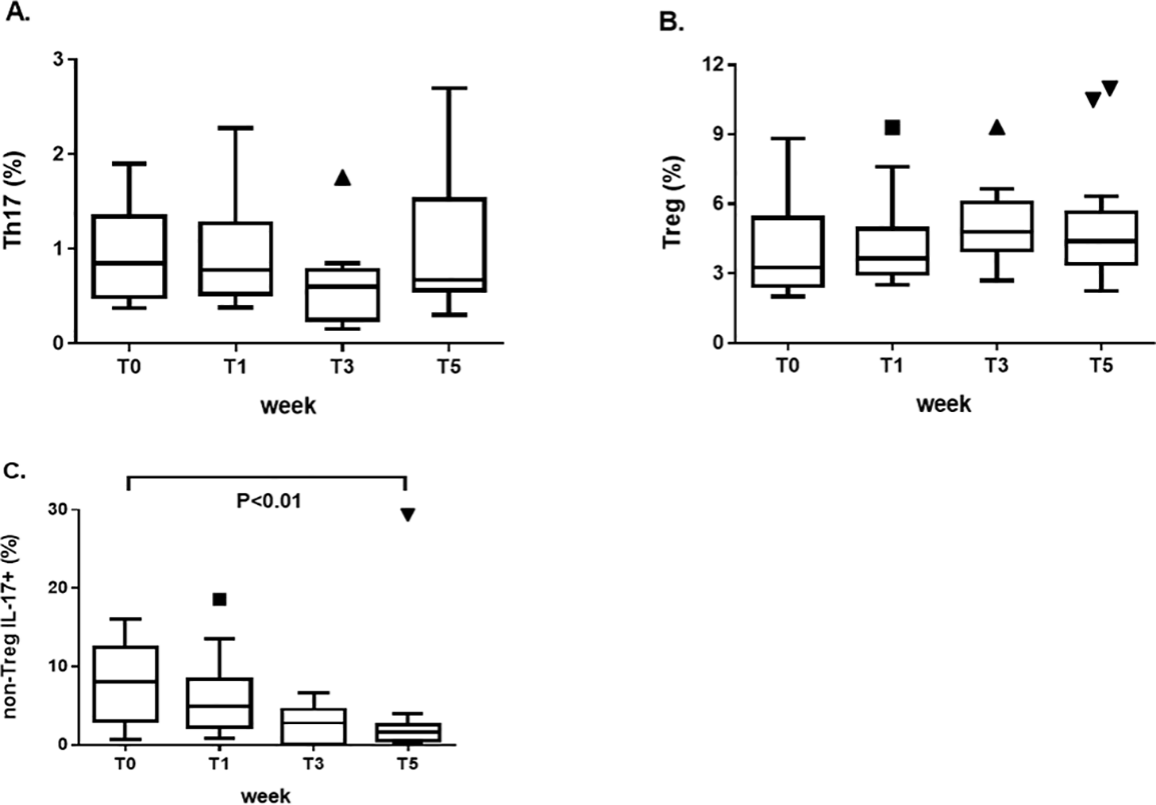
Change in the frequencies of Th17 and Treg cells (including the nTreg, eTreg, and non-Treg fractions) in the peripheral blood of patients with rheumatoid arthritis at the main timepoints of the study set at T0 (baseline, before starting the low-sodium dietary regimen), T1 (after 1 week, at the end of the “run-in” period of low-sodium dietary regimen), T3 (after 3 weeks, at the end of low-sodium dietary regimen), and T5 (at the end of the study, after 2 weeks of normal sodium dietary regimen). (A) No significant change in the frequencies of Th17 cells between the main timepoints of the study. (B) No significant change in the frequencies of Treg cells between the main timepoints of the study. (C) The frequency of non-Treg fraction of Treg cells (exhibiting the capability to trans-differentiate into Th17 cells and indeed being positive for the intracellular staining with IL-17 mAb) significantly decreased at the end of the study with respect to baseline. Data are shown as Tukey boxplots; lines represent the median level with 25^th^-75^th^ percentile; data not included between the whiskers are plotted as outliers with dots.

We investigated the change in the frequencies of Th17 and Treg cells (including the nTreg, eTreg, and non-Treg fractions) in the peripheral blood of patients with RA and SLE following a dietary regimen characterized by the modulation of sodium intake. As shown in Fig 3A, we did not find any significant change in the frequencies of Th17 cells in RA patients, although a clear trend toward a reduction over the low-sodium dietary regimen followed by an increase at the end of the study was observed. Similarly, no significant change was observed in the frequencies of Treg cells, despite a trend toward an increase during the low-sodium intake with respect to baseline and a decrease at the end of normal sodium regimen (Fig 3B). Among the different subtypes of Treg cells, the frequency of non-Treg fraction producing IL-17 significantly decreased at the end of the study with respect to baseline (8.1/3.04–12.4 vs 1.6/0.6–2.5; p<0.01) (Fig 3C).

**Fig 4:**
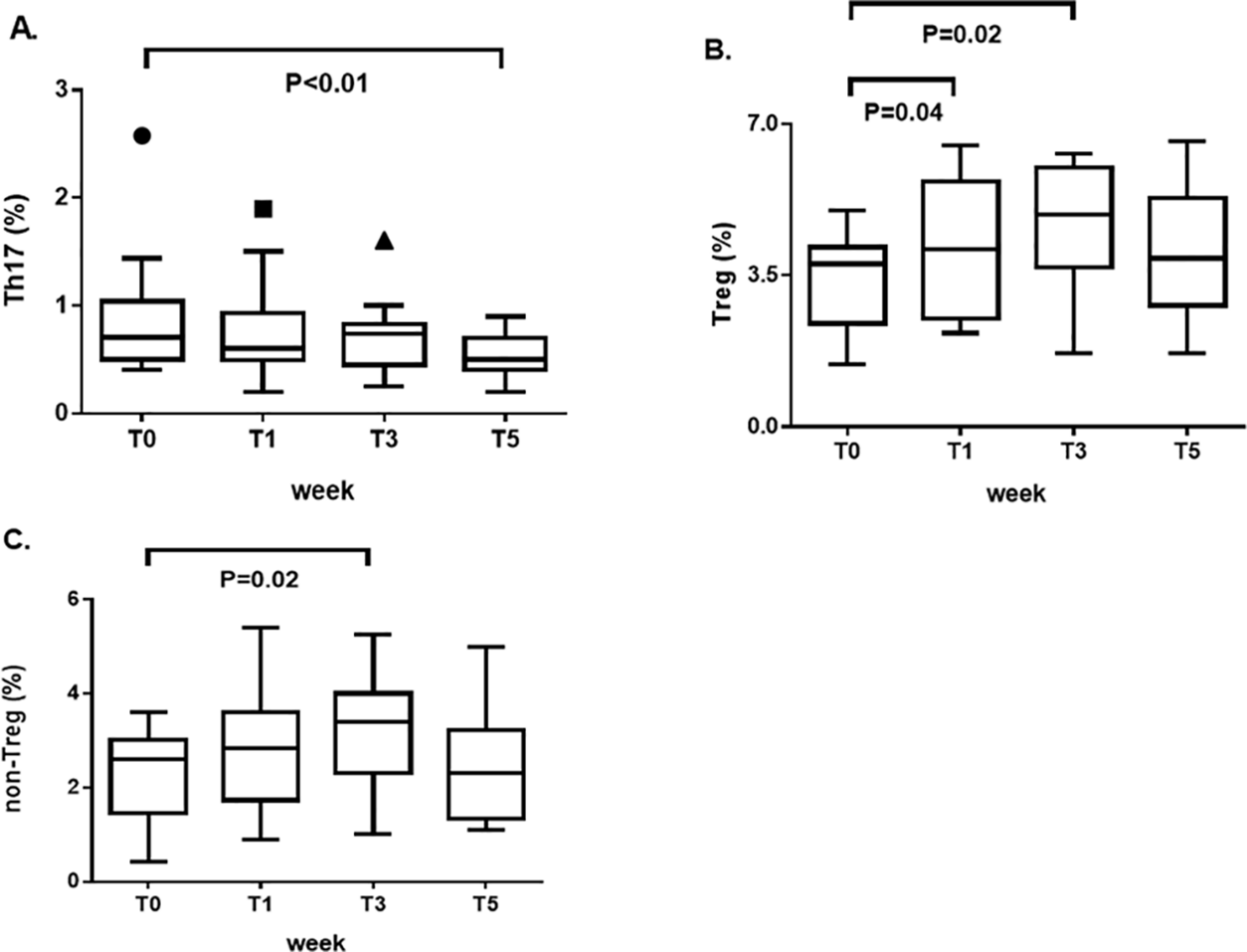
Change in the frequencies of Th17 and Treg cells (including the nTreg, eTreg, and non-Treg fractions) in the peripheral blood of patients with systemic lupus erythematosus at the main timepoints of the study set at T0 (baseline, before starting the low-sodium dietary regimen), T1 (after 1 week, at the end of the “run-in” period of low-sodium dietary regimen), T3 (after 3 weeks, at the end of low-sodium dietary regimen), and T5 (at the end of the study, after 2 weeks of normal sodium dietary regimen). (A) Reduction in the percentage of Th17 cells at T5 compared to T0. (B) Significant increase in the percentage of Treg cells at both T1 and T3 compared to T0. (C) Significant increase in the percentage of non-Treg cells at T3 compared to T0. Data are shown as Tukey boxplots; lines represent the median level with 25^th^-75^th^ percentile; data not included between the whiskers are plotted as outliers with dots.

In SLE patients, we found a progressive reduction in the percentage of Th17 cells that reached a statistical significance at T5 (0.5/0.4–0.7) compared to T0 (0.7/0.5–1; p = 0.01) (Fig 4A), and a significant increase in the percentage of Treg cells following the low-sodium dietary regimen at both T1 (4.1/2.5–5.7) and T3 (4.9/3.7–6) compared to T0 (3.7/2.4–4.1; p = 0.04 and p = 0.02, respectively) (Fig 4B). Examining the different fractions of Treg cells, a significant increase in the percentage of non-Treg cells at T3 (3.4/2.3–4) compared to T0 (2.6/1.5–3; p = 0.02) was found, as shown in Fig 4C.

### Detection of PBMC apoptosis and lymphocytes proliferation

Spontaneous apoptosis of PBMCs was evaluated at the main timepoints of the study. Neither in RA nor in SLE patients we found any significant apoptosis or proliferation modulation during the two dietary sodium regimens (not shown).

### Levels of serum cytokines

**Fig 5:**
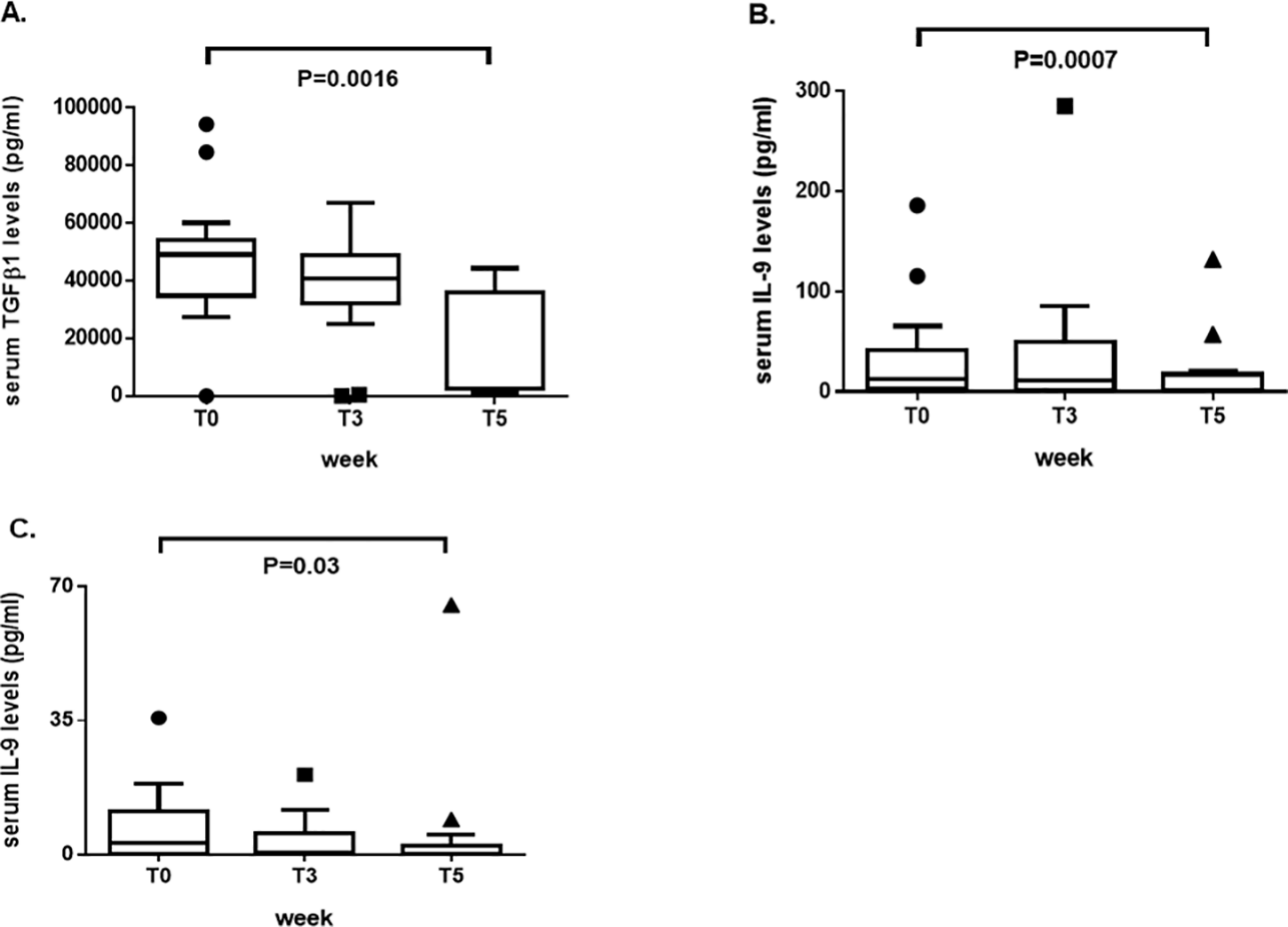
Levels (pg/ml) of pro-inflammatory/immunoregulatory cytokines measured in the serum of patients with RA and SLE at the main timepoints of the study set at T0 (baseline, before starting the low-sodium dietary regimen), T1 (after 1 week, at the end of the “run-in” period of low-sodium dietary regimen), T3 (after 3 weeks, at the end of low-sodium dietary regimen), and T5 (at the end of the study, after 2 weeks of normal sodium dietary regimen). (A) Significant reduction in serum TGFβ1 levels and (B) IL-9 levels at T5 compared to T0 in patients with rheumatoid arthritis. (C) Significant reduction in serum IL-9 levels in patients with systemic lupus erythematosus at T5 with respect to T0. Data are shown as Tukey boxplots; lines represent the median level with 25^th^-75^th^ percentile; data not included between the whiskers are plotted as outliers with dots.

Levels of pro-inflammatory/immunoregulatory cytokines involved in the pathogenesis of RA and SLE were analyzed to find possible changes alongside dietary sodium intake. In RA patients, we found a significant reduction at T5 compared to T0 in serum levels (pg/ml) of both TGFβ1 (3002/2193-37069 vs 49069/38221-58005; p = 0.0016) and IL-9 (1.65/0-17.5 vs 12.8/3-41.2; p = 0.0007) (Fig 5A and 5B, respectively). Serum IL-9 levels were also reduced in SLE patients at T5 with respect to T0 (0/0-2.25 vs 2.3/0-11.2; p = 0.03) as shown in Fig 5C. No significant differences were observed in the other cytokines measured (not shown).

## Discussion

To our knowledge, this is the first study investigating the effects of dietary sodium intake on adaptive immune response in patients with autoimmune diseases.

Patients with RA and SLE accepted to undergo a five-week period of a composite dietary regimen starting with a restricted daily sodium intake followed by a normal-sodium daily intake. RA and SLE are multifactorial diseases whose genetic and environmental factors are continually being explored [1].

Among environmental factors, dietary regimen is receiving growing attention, due to the capability of affecting inflammation, antigen presentation, antioxidant defense mechanisms, allergies and gut microbiota [29]. Furthermore, modest increases in the concentration of sodium chloride markedly enhanced pro-inflammatory Th17 responses in vitro, as did a high-sodium diet in vivo, in 2 independent studies in murine models of autoimmune diseases [3,4]. These studies also provide possible hints on the molecular mechanisms by which sodium chloride may sustain the inflammatory responses. In the former, a modest increase in NaCl concentration could stimulate an almost logarithmic in vitro induction of IL-17A in naïve CD4+ cells mediated through p38/MAPK, nuclear factor of activated T cells 5 (NFAT5) and serum/glucocorticoid-regulated kinase 1 (SGK1); importantly, the addition of 40 mM of NaCl to Th17 differentiaton cultures not only increased IL-17A expression but also led to a pathogenic phenotype of Th17 cells [3]. Likewise, in the latter study, a modest increase in salt concentration induced SGK1 expression, promoting IL-23R expression and enhancing Th17 cell differentiation in vitro and in vivo [4].

Since then, studies in humans with RA and MS proved further consistency on the role of excess sodium intake in favouring the development or exacerbation of these diseases, although no biological effect was tested [6,7]. Yet, the issue is of utmost importance, since salt content in processed foods and ‘fast food’ largely consumed in the Western countries can be more than 100 times higher in comparison to similar homemade meals [30]. Notably, there is a high prevalence of autoimmune diseases in Western societies than in the Eastern world and developing countries [31,32]. Also, the more recent European guidelines on cardiovascular disease prevention reaffirm the recommendation to not exceed 5 grams of salt per day [18]. This was the threshold of sodium intake for the patients enrolled in our study in the normal sodium phase, which was preceded by a dietary sodium restriction. Adherence to the dietary regimen was demonstrated by the levels of 24-hour urinary sodium excretion which were widely lower than the established cut-off of 85 mEq/24h at scheduled timepoints to ascertain the restricted sodium intake in the first 3 weeks. Interestingly, the levels of 24-hour urinary sodium excretion at baseline (median/25°-75° percentile) were high (113.5/84.9–165.5 and 153.5/96.3–215 for RA and SLE patients, respectively), confirming that in most Western countries sodium intake usually exceed the recommended limit [18].

The adoption of healthier dietary habits with low sodium intake is known to influence cardiovascular risk [33] and gastric cancer [34]; our study shows also evidence of an anti-inflammatory effect in autoimmune diseases. The finding was overt in patients with SLE, who exhibited a progressive reduction in the percentage of the pro-inflammatory Th17 cells over the 5 weeks of the study and a parallel increase in the percentage of Treg cells following the low-sodium dietary regimen. A similar change in the frequencies of Th17 cells and Treg cells was observed in patients with RA, although the comparisons between the different timepoints did not reach a statistical significance. This is the first demonstration of the induction of biological effects of sodium on adaptive immunity ex vivo in patients with autoimmune diseases, while a modulation of macrophages which resulted skewed toward a pro-inflammatory [35,36] or an anti-inflammatory phenotype and function had already been reported [37].

Another data supporting a beneficial effect a low-sodium dietary regimen derives by the observation that IL-9 serum levels in RA and SLE patients significantly decreased from baseline to the end of the study. This is in line with previous findings in vitro, demonstrating that high sodium chloride concentration led Th17 to display a pathogenic phenotype, characterized by the expression of pro-inflammatory cytokines, including IL-9 [3]. This cytokine is produced by activated T cells and supports the growth of Th but not cytolytic clones and has pleiotropic functions on the immune system [38,39]. Recent studies provided evidence of a pro-inflammatory role of IL-9 in RA patients, where IL-9 and Th9 cells were overexpressed in synovial tissue and correlated with the degree of histological organization of B and T cells in ectopic lymphoid structures [40]. Furthermore, peripheral blood Th9 cells were increased in patients with established and early untreated RA with respect to healthy controls, and expanded after in vitro exposure to citrullinated aggrecan peptide [40]. In lupus murine models, an expansion of Th9 cells in the spleens of lupus-prone mice was demonstrated and also serum IL-9 levels were elevated, which were positively correlated to anti-dsDNA antibody titer [41]. Although in SLE patients higher IL-9 serum levels than controls were observed, no associations with disease activity, chronic damage and clinical features were found [42]. Notably, TGFβ1, a regulatory cytokine that affects proliferation, differentiation, and survival of several types of cells, promotes the redirection of naÏve T cells from a Th2 to Th9 cell differentiation pathway [43] and was abundantly seen in most actively proliferating synovial intimal cells from patients with RA [44]. Furthermore, while a protective effect of TGFβ1 in collagen-induced arthritis (CIA) was demonstrated [45], other evidences showed that the local inhibition of TGFβ1 by a neutralizing antibody suppressed acute and chronic arthritis in an experimental model of chronic erosive polyarthritis [46]. These findings suggest that the pleiotropic effects of TGFβ are context-related and, indeed, in our RA patients the sodium restriction induced a dramatic reduction of TGFβ1 serum levels in a context of reduced inflammatory burden.

In conclusion, this is the first study investigating the effects of cooking salt in adaptive immunity ex vivo in patients with RA and SLE, suggesting that a restricted sodium dietary intake could contribute to dampen the pro-inflammatory response. Our results add information on a potential new modifiable environmental factor in autoimmune diseases; however, due to the limited sample size, further studies are encouraged to define the utility and modality of dietary habits to ameliorate the outcome in these patients.

## Supporting information

S1 File Recommendations for patients to follow the low-sodium dietary regimen. (DOCX)
