# Supplementary material for: The role of dietary sodium intake on the modulation of T helper 17 cells and regulatory T cells in patients with rheumatoid arthritis and systemic lupus erythematosus
Source: PLoS One. 2017 Sep 6;12(9):e0184449. doi: 10.1371/journal.pone.0184449 (PMC5587319; doi:10.1371/journal.pone.0184449)
Supplement: S1 File — (DOCX) [file pone.0184449.s001.docx]

**Low-sodium diet: this kind of diet requires the limitation of high-sodium foods and the elimination of cooking salt.**

**General principles:**

Do not modify the amount of what you usually eat, even though you should be aware of substituting not recommended food, keeping stable your weight, varying the amount of food and following these general principles:

- Avoid condiments such as cube, soy sauce, mustard, which contains sodium and monosodium glutamate used to enhance the flavour of products
- Prefer condiments such as lemon, aromatic herbs and vinegar
- Eat preferably fresh and handmade food and avoid canned food or preserves which contain more sodium
- Do not add salt
- Prefer healthy cooking such as steam or grill and use raw oil to add flavour
- Drink a lot of water, but low-sodium water, eat lots of fruit and vegetables

**Recommended Foods**

- Meat: low-fat meat, such as chicken, turkey, calf and horse are allowed
- Dairy products: low-fat milk, yogurt and light cheese such as ricotta and mozzarella are allowed.
- Fish: sea-fish such as codfish, hake, gilthead bream, river fish such as carp and trout are allowed.
- Cereals: eat bread without salt, low-salt baked products, rice, barley and oat. You can eat also potatoes but not french fries.
- You can eat boiled and poached eggs.
- You can eat fresh but not canned legumes.

Fruit and vegetables (except for those listed below) are allowed.

**Not recommended foods**

- Lunch meat, cured meat
- Full-fat milk, yogurt and aged cheese such as pecorino and gorgonzola.
- Fat fish such as eel and sea-fish, shellfish, and preserved fish (fish eggs, marinated and smoked fish).
- Cereals: every baked products, sweets, pizza and bread.
- Fruit and vegetables: dried fruit, some vegetables such as spinach, fennel, carrots, celery and artichoke.
- Condiments: avoid soy sauce, ketchup, mayo, margarine, mustard, canned tomato sauce, butter, cube.

**Recommended condiments:**

Extra-virgin olive oil (no more than one spoon at lunch and one at dinner), aromatic herbs, lemon and vinegar.

DO NOT ADD SALT TO FOOD.
